# Factors associated with use of malaria control interventions by pregnant women in Buwunga subcounty, Bugiri District

**DOI:** 10.1186/s12936-016-1407-2

**Published:** 2016-07-04

**Authors:** Elizabeth Muhumuza, Noel Namuhani, Bonny Enock Balugaba, Jessica Namata, Elizabeth Ekirapa Kiracho

**Affiliations:** School of Public Health, College of Health sciences, Makerere University, P.O. Box 7072, Kampala, Uganda; Department of Health Policy Planning and Management, School of Public Health, Makerere University, Kampala, Uganda

**Keywords:** ITN use, IPTp2 uptake, Malaria control interventions, Pregnant women

## Abstract

**Background:**

In Uganda, the Government has promoted the use of intermittent preventive treatment of malaria in pregnancy (IPTp) and insecticide-treated bed nets (ITNs) as malaria control strategies for pregnant women. However, their utilization among pregnant women is low. This study aimed at assessing factors associated with use of IPTp for malaria and ITNs by pregnant women in Buwunga sub-county, Bugiri District.

**Methods:**

This was a cross-sectional study, conducted in Buwunga sub-county, Bugiri District, employing quantitative data collection tools. A total of 350 household members were randomly selected to participate in the study. Data were entered and analysed using Epi info version 3.5.1; bivariable and multivariable analysis was done to assess the factors associated with use of IPTp and ITNs among pregnant women.

**Results:**

The level of uptake of IPTp1 (at least one dose) was 63.7 % while IPTp2 (at least two doses) was 42.0 %. More than half (58.6 %) of the mothers had slept under an ITN the night before the survey. Slightly more than half (51.9 %) of the mothers mentioned stock outs as the major reason for not accessing IPTp and ITNs. The main factors that were statistically significant for IPTp2 uptake were the knowledge of mothers on IPTp2 (AOR 2.48 95 % CI 1.53–4.02) and providing women with free clean water at the antenatal care (ANC) clinic (AOR 3.63 95 % CI 2.06–6.39). Factors that were significant for ITN utilization included education level of mothers (AOR 2.03 95 % CI 1.09–3.78), ease of access (AOR 2.74 95 % CI 1.65–4.52), and parity (AOR 1.71 95 % CI 1.01–1.29).

**Conclusion:**

The level of uptake of the two recommended doses of sulfadoxine–pyrimethamine (SP) tablets for malaria prevention (IPTp2) was low, slightly more than half of the mothers slept under an ITN the night before the survey. Appropriate measures to increase the level of uptake of IPTp2 and coverage of ITNs among pregnant women should be implemented, and these include providing health education about IPTp and ITNs, and ensuring that mothers are provided with free safe clean water at ANC clinic.

## Background

Malaria kills over a million people each year and causes 300–500 million clinical cases worldwide, with 80 % of the deaths and 91 % of the clinical cases occurring in sub-Saharan Africa [1]. More than half of the world’s population is at risk of contracting malaria [2]. Malaria disproportionately affects children and pregnant women, especially in resource-limited, developing countries [2]. During pregnancy, malaria may lead to several complications that include premature delivery or low birth weight, cerebral malaria with abnormal behaviour, impairment of consciousness, seizures, coma, or other neurological abnormalities [3].

The World Health Organization (WHO) recommends a package of intermittent preventive treatment in pregnancy (IPTp) with sulfadoxine–pyrimethamine (SP) and use of insecticide-treated bed nets (ITNs), together with effective case management of clinical malaria and anaemia in malaria-endemic areas, such as Africa [4, 5]. IPTp consists of two doses of SP taken one month apart commencing in the second trimester. With collaboration from malaria and reproductive health programmes in Uganda, both IPTp and ITNs are usually availed at the ANC clinics [6]. By 2010, the Roll back malaria partnership had aimed at ensuring that all pregnant women receive at least two doses of IPTp and 80.0 % of people at risk of suffering from malaria sleep under ITNs [7]. However the coverage of these preventive interventions among pregnant women remains low for many countries in sub-Saharan Africa [8]. The mean coverage of two doses of SP is 24.5 % despite having over 80 % of the women attending at least two ANC visits according to a survey in 27 countries [7]. This is an indication of substantial missed opportunities at the ANC clinics [6]. The same survey indicated that only 35.3 % of pregnant women slept under ITNs.

According to the 2009 Uganda Malaria Indicator Survey, 44 % of pregnant women had slept under an ITN the night before the survey and 32 % received two doses of IPTp [9]. This fell short of the Ministry of Health target of increasing IPTp2 (at least two doses) uptake to 80 % (Health Sector Strategic Plan II). The situation is worse in Bugiri District. According to Bugiri District STRIDES Baseline Needs Assessment, only 30.2 % of the pregnant women acquired more than one dose of IPTp and only 40 % slept under an ITN. This low coverage is said to have escalated the level of malaria morbidity and mortality among pregnant women and children under the age of 5 years.

High access and utilization of IPTp and ITNs by pregnant women is necessary to reduce the burden of malaria among pregnant women. The study examined the factors associated with the use of these malaria control interventions by pregnant women in Buwunga sub-county, Bugiri District.

## Methods

### Study design and setting

This was a community-based, cross-sectional, household study that utilized quantitative data collection tools. The study was conducted in Buwunga sub-county, Bugiri District, which is located in southeastern Uganda. The District borders Tororo to the northeast, Iganga to the west, Namutumba to the northwest, Mayuge to the southwest, and Namayingo to the east. It is 178 km from the capital city of Kampala. The district has a population of 390,076 [10]. Buwunga sub-county is located in the northwestern part of Bugiri District with a population of 46,979 [10]. It has four parishes and 28 villages. Buwunga has one health centre III and three health centre II. The study population comprised all pregnant women in the study area, as well as women who had given birth 12 months prior to the study.

### Sample size determination and sampling

A sample size of 350 households was determined using the Kish Lisle formula of 1965. The calculation was based on a 95 % confidence interval, a 35 % estimated proportion of mothers who use malaria control interventions in Bugiri District [9], a sampling error of 5 % and a non-response rate of 10 %. Multistage sampling was used. The stages involved simple random sampling of three parishes out of the four parishes in the Sub-county, followed by the selection of four villages out of the six to eight villages per selected parish. The random selection of the parishes and the villages involved use of the ballot method. Lastly, 29 households were randomly sampled from each of the 12 villages. Village health team members (VHTs) and local leaders helped in locating homes with eligible respondents. A list of all eligible households in each village was obtained, and then systematic random sampling was used to obtain the 29 households from each village. The sampling interval depended on the number of eligible households in each village.

### Data collection and analysis

Semi-structured questionnaires were used to collect information on IPTp2 uptake and utilization of ITNs, women’s sociodemographic factors, women’s knowledge and attitudes towards ITNs and ITPp, and health system factors. The tools were pre-tested and corrected on the basis of the findings to ensure that they were accurate and comprehensive. Data was collected during a dry season in the month of September, 2014. Quantitative data were entered and analysed using EPI INFO Version 3.5.1 computer software. Descriptive statistics, such as frequencies and proportions, were used to describe the study subjects. Bivariable analysis and multivariable analysis was done to obtain odds ratios and adjusted odds ratios, respectively, at 95 % confidence intervals to determine the factors associated with use of ITNs and IPTp2. Variables that were statistically significant at bivariable analysis (P = <0.05) were considered for multivariable analysis.

## Results

### Sociodemographic characteristics of the respondents

The majority (61.7 %) of the women were more than 30 years of age, 17.4 % were teenagers. The mean age of the respondents was 26.2 years. Most (80.9 %) of the women had attained at most primary education. Some (58.0 %) were Muslims, and 25.4 % were Anglicans (25.4 %). Some 86.9 % were married; 85.4 % of the women were farmers. More than half (52.6 %) of the women had had four or more pregnancies (Table 1).

**Table 1:** Sociodemographic characteristics of the mothers

Variable	Frequency (n = 350)	Percentage (%)
*Age* (years)		
<20	61	17.4
20–30	73	20.9
>30	216	61.7
*Highest level of education*		
None	51	14.6
Primary	232	66.3
Secondary	65	18.6
Tertiary	2	0.6
*Religion*		
Anglican	89	25.4
Catholics	31	8.9
Moslem	203	58.0
Others^a^	27	7.7
*Marital status*		
Married/cohabiting	304	86.9
Separated	14	4.0
Single	30	8.6
Widowed	2	0.6
*Occupation*		
Farmer	299	85.4
Not employed	27	7.7
Others^b^	24	6.9
*Parity*		
One	61	17.4
Two	52	14.9
Three	53	15.1
Four	49	14.0
Five and above	135	38.6
*Number of co*-*wives*		
0	222	63.4
1	89	25.4
>1	39	11.2

Others^a^ Born-again Christian, Seventh Day Adventist, Atheist

Others^b^ Civil servant, Business, Commercial motorcyclist

### Uptake of IPTp at ANC clinics

The level of uptake of IPTp1 (at least one dose) was 63.7 % while IPTp2 was 42.0 %. Most respondents (63.1 %) first attended ANC clinic in their second trimester of pregnancy while 10.9 % first attended ANC clinic in their final trimester.

### Utilization of ITNs

More than half (58.6 %) of the mothers had slept under an ITN the night before the survey; 82.9 % of the respondents had used the net for at least 1 year. The health facility (35.4 %) and sub-county (43.7 %) headquarters were the main sources of the nets; 17.5 and 3.4 % of the mothers obtained their ITNs from the market and non-governmental organizations (NGOs), respectively. The nets that were obtained from the health facility, sub-county headquarters, and NGOs were free of charge.

### Individual factors

About half (53.1 %) of the mothers mentioned that they knew the purpose of IPTp tablets; 50.3 % mentioned the correct use of IPTp tablets as a malaria preventive remedy. The majority (88.6 %) of the mothers believed that they were susceptible to malaria; 54.0 % said that a mother had to take the tablets three or more times; 33.4 % confessed having no idea of the number of times to take the SP tablets. Over 47 % of the mothers did not know the effects of malaria on pregnant women; when asked about the effect of malaria on their unborn child, most mothers (45.1 %) did not know of any effect, and 39.4 % mentioned abortion. The major source of knowledge about IPTp was from health workers (88.2 %), friends (4.8 %) and the media (4.8 %). Mothers who mentioned that SP tablets had to be taken at least twice and that the tablets are for malaria prevention during pregnancy were considered to be knowledgeable about the use of IPTp (Table 2).

**Table 2:** Individual and healthcare system factors for use of IPTp and ITNs

Variable	Frequency (n = 350)	Percentage (%)
*Knows the use of IPTp*		
Yes	186	53.1
*Use of IPTp tablets (n* = *186)*		
Prevent malaria	95	50.3
Promotes weight gain	14	7.4
Gives a lot of blood	42	22.2
Makes the baby strong and healthy	23	13.8
Others	12	6.3
*Number of times a pregnant woman has to take IPTp tablets*		
Once	13	3.7
At least twice	220	62.9
Do not know	117	33.4
*Effects of malaria on pregnant women*		
Anaemia	100	28.6
Death	42	12.0
Do not know	165	47.1
Nothing	2	0.6
Others	41	11.7
*Effects of malaria on unborn baby*		
Abortion	138	39.4
Intra-uterine death	24	6.9
Low birth weight	18	5.1
Do not know	158	45.1
Others	12	3.4
*Reasons for not accessing ITNS/IPTp with ease (n* = *239)*		
Long waiting time	46	19.2
Nurses do not attend to patients—better to do each separately well	22	9.2
Stock-outs of tablets and ITNs	124	51.9
Demand for money by health workers	34	14.2
Others	13	5.4

### Healthcare system factors

Most (68.6 %) of the mothers were served with free clean water at the ANC clinic to swallow the tablets; 88.0 % said that health workers had a positive attitude towards pregnant women; 82.9 % of the respondents spent more than 30 min at ANC clinic; 54.9 % said that the ANC clinic was far away (>5 km) from their homes. The majority (64.0 %) of the women said that it was difficult to access ITNs; 81.7 % said it was very easy to access IPTp tablets at ANC clinics. Some of the commonly cited reasons for not accessing ITNs and IPTp by pregnant mothers included shortage of tablets and ITNs (51.9 %), long waiting time (19.2 %), and demand for money by health workers (14.2 %) (Table 2).

### Factors associated with use of IPTp2

Being knowledgeable about the use of IPTp tablets (OR 2.75, 95 % CI 1.76–4.28), being in position to easily access IPTp tablets (OR 2.10, 95 % CI 1.16–3.89), and being served with free clean water (OR 4.35, 95 % CI 2.56–7.40), at the ANC clinic were factors that showed a statistically significant association with the uptake of IPTp2 at bivariable analysis level. Women who were knowledgeable about the use of IPTp were 2.75 times more likely to receive at least two doses of IPTp tablets compared to those women with less knowledge. Serving mothers with free clean water at ANC clinics increased the likelihood of mothers receiving at least two doses of IPTp tablets by 4.35 times (Table 3).

**Table 3:** Bivariable and multivariable analysis of factors associated with IPTp2 uptake

Variable	Category	IPTp2 uptake frequency (%)		COR (95 % CI)	AOR (95 % CI)
		Yes (n = 147)	No (n = 203)		
Education	Primary and below	115 (78.2)	168 (82.8)	0.75 (0.44–1.29)	
	Above primary	32 (21.8)	35 (17.2)	1.0	
Marital status	Married	127 (86.4)	24 (87.2)	0.93 (0.50–1.74)	
	Not married	20 (13.6)	26 (12.8)	1.0	
Parity	<3	44 (29.9)	69 (34.0)	1.0	
	>5	41 (27.9)	56 (27.6)	1.15 (0.66–2.01)	
	3–5	62 (44.3)	78 (55.7)	1.24 (0.75–2.00)	
Employment	Farmer	129 (87.8)	177 (83.7)	1.39 (0.75–2.58)	
	Other jobs^a^	18 (12.2)	33 (16.3)	1.0	
Distance to health facility (km)	>5	75 (51.0)	117 (57.6)	0.77 (0.50–1.17)	
	<5	72 (49.0)	86 (42.4)	1.0	
Knowledgeable on IPTp use	Yes	51 (40.5)	44 (19.6)	2.75 (1.76–4.28)**	2.48 (1.53–4.02)**
	No	75 (59.5)	180 (80.4)	1.0	
Ease of access of IPTp	Yes	129 (87.8)	157 (77.3)	2.10 (1.16–3.80)**	1.71(0.89–3.30)
	No	18 (12.2)	46 (22.7)	1.0	
Perceived susceptibility to malaria	Yes	135 (91.8)	175 (86.2)	1.80 (0.88–3.67)	
	No	12 (8.2)	28 (13.8)	1.0	
Served with water	Yes	125 (85.0)	115 (56.7)	4.35 (2.56–7.40)**	3.63 (2.06–6.39)**
	No	22 (15.0)	88 (43.3)	1.0	

*COR* Crude odds ratio, *AOR* Adjusted odds ratio, *CI* Confidence interval

* p < 0.05, ** p ≤ 0.01

^a^Other jobs include civil servants, business and traditional healer

Variables that were statistically significant for use of malaria control interventions at bivariable analysis were considered for multivariable analysis. The logistic regression analysis revealed that being knowledgeable of IPTp use increased the likelihood of receiving at least two doses of IPTp tablets by 2.48 times, (AOR 2.48, 95 % CI 1.53–4.02). Providing free clean water at ANC clinics increased the chances of women taking at least two doses of IPTp tablets by 3.63 times (AOR 3.63, 95 % CI 2.06–6.39) (Table 3).

Women who had education level of primary and below had reduced chances of sleeping under a mosquito net (AOR 0.49, 95 % CI 0.26–0.92). Women who had three to five pregnancies had increased chances of having slept under a net compared to women who had fewer than three and more than five pregnancies. Long distance to the health facility reduced chances of women sleeping under a net by 60 % (Table 4). Multivariable analysis also revealed that education level, ease of access and parity remained significant for utilization of bed nets. Women who had attained an education level of secondary and above were two times more likely to sleep under ITNs compared to those with an education level below secondary (AOR 2.03, 95 % CI 1.09–3.78) (Table 4).

**Table 4:** Bivariable and multivariable analysis of factors associated with ITN use

Variable	Category	ITN use frequency (%)		COR (95 % CI)	AOR (95 % CI)
		Yes (n = 205)	No (n = 145)		
Education	Primary and below	156 (76.1)	127 (87.6)	0.45 (0.25–0.81)**	0.49 (0.26–0.92)*
	Above primary	49 (23.9)	18 (12.4)	1.0	
Parity	<3	60 (29.3)	53 (36.6)	1.0	
	>5	53 (25.9)	44 (30.3)	1.06 (0.61–1.83)	1.20 (0.68–2.14)
	3–5	92 (44.9)	48 (33.1)	1.69 (1.02–2.81)*	1.71 (1.01–1.29)*
Age (years)	<20	35 (17.1)	26 (17.9)	1.0	
	>30	37 (18.0)	36 (24.8)	0.76 (0.39–1.51)	
	20–30	133 (64.9)	83 (57.2)	1.19 (0.67–2.11)	
Distance to health facility (km)	>5	102 (49.8)	90 (62.1)	0.60 (0.39–0.93)*	0.83 (0.52–1.34)
	<5	103 (49.0)	55 (37.9)	1.0	
Time spent at ANC clinic (h)	<1	26 (17.7)	34 (16.7)	1.06 (0.60–1.87)	
	>1	121 (82.3)	169 (83.3)	1.0	
Ease of access of ITN	Yes	94 (45.9)	32 (23.1)	2.99 (1.85–4.82)**	2.74 (1.65–4.52)**
	No	111 (54.1)	113 (77.9)	1.0	

*COR* Crude odds ratio, *AOR* Adjusted odds ratio, *CI* Confidence Interval

* p < 0.05, ** p ≤ 0.01

## Discussion

The WHO recommendations emphasize that pregnant women should receive at least two doses of SP during pregnancy. The 42.0 % uptake of SP for IPTp2 in this study is lower than the targeted global coverage of 80 % in 2010 [2] and it is also lower than the national target of 85 % [11]. These findings are similar to those of a study conducted in Luweero which revealed that only 35.8 % of the mothers had received two or more doses of IPTp [12]. Lower uptake was reported in Nigeria where only 6.5 % of pregnant women had taken the recommended two doses of SP during pregnancy [13]. This low level of uptake of IPTp2 could be due to the reported regular stock-outs of SP tablets, which is one of the major barriers to the IPTp2 supply at health facilities in Uganda [14]. This finding implies that there is a need for regular supply of SP tablets in health facilities.

Bed net usage in this study was slightly higher than the national coverage of 47 % [15] and higher than the 44 % from the Uganda malaria indicator survey [9]. However, ITN use in this study was low compared to the national target of 85 % [16]. ITN utilization was low probably because most of the mothers said that it was hard to access ITNs. Although these nets are sometimes given out at health facilities, distribution centres are far from mothers, as reported by respondents.

In this study, about half of the respondents were knowledgeable about the use of IPTp tablets. Mothers who were knowledgeable about IPTp use were 2.48 times more likely to have received at least two doses of IPTp tablets (AOR 2.48, 95 % CI 1.53–4.02). These findings are in agreement with findings of a study conducted in Tanzania which showed that there was generally high knowledge of IPTp among pregnant women and that this high knowledge correlated well with IPTp coverage [17]. However, having knowledge alone is not enough to translate into IPTp uptake, as demonstrated in this study. Although 62.9 % of the respondents knew that SP should be taken at least twice, only 42.0 % actually received the recommended two doses.

The study findings also corroborated with findings of a study done in Korogwe District, northeastern Tanzania [18] which showed that although the majority recognized that SP prescribed at ANC facilities was for malaria preventive purposes, only 38.0 % received two doses of SP tablets. This clearly demonstrates the knowledge gap among ANC clients and it shows that interventions that can aid in transformation of knowledge into practice are needed.

It was revealed that women who had attained post-primary education level were two times more likely to have slept under ITNs than women of pre-secondary education level. This was probably because educated women had good knowledge about the dangers of malaria, so they developed the need to protect themselves by sleeping under mosquito nets. These study findings were not in agreement with study findings in Sudan which showed that although the level of education played a major role in access to curative and preventive health services, there was no significant association between the level of education and ITN usage [19], which was consistent with study findings by [20] in Tanzania where the level of education of the household heads significantly influenced the use of nets. The study findings therefore imply that there is need to package information use of ITNs to target those mothers who lack formal education.

Mothers who had had three to five pregnancies had increased chances of sleeping under ITNs (AOR 1.69, P < 0.05) than mothers who had had fewer than three and more than five pregnancies. This is probably because mothers who had fewer than three pregnancies had inadequate knowledge about the dangers of malaria, while those mothers with more than five pregnancies had a lower perceived need to visit ANC clinic because of their parity, and so they were less likely to go to ANC clinic, and subsequently had the reduced chance of accessing ITNs. This was in agreement with study findings in Sudan which showed that the increasing number of deliveries inversely related to the likelihood of ITN usage (AOR 0.1, P < 0.05), [19]. This implies that there is need to educate mothers and encourage them to attend ANC clinic and use bed nets no matter how many pregnancies they have had.

### Healthcare system factors associated with use of malaria control interventions

Safe, clean water was available to the respondents for taking SP at ANC clinics. This was confirmed by 68.6 % of mothers who said they were provided with free water to swallow the tablets at the clinic. Availability of water at ANC clinics encourages direct observation by nurses to ensure that women swallow the medicine, which increases the uptake of IPTp. Indeed, providing women with free clean water at ANC clinics increased the chance of women taking at least two doses of IPTp tablets by 3.63 times. This was contrary to study findings in Ghana where provision of safe water was not significantly associated with the number of SP doses received by respondents (OR 0.56, CI 0.19–1.70) [21]. These study findings therefore imply that there is need to increase access of safe water to all mothers at ANC clinics, so as to increase the level of IPTp2 uptake.

Stock-outs of SP tablets was one of the factors for low uptake of IPTp. Studies done in Kilombero, southwestern Tanzania [22] and Kibaha, eastern Tanzania [23], indicated that coverage of IPTp is influenced more by the availability of SP tablets. In a study conducted in Bungoma, eastern Kenya, SP tablets for IPTp were not always available in most health facilities and this was said to have contributed to the low uptake of IPTp2 in the district [24]. This implies that there is need to ensure a constant supply of SP tablets at health facilities to increase the coverage of IPTp2.

The study revealed that mothers who mentioned that it was easy to access ITNs were about three times more likely to have slept under an ITN the night before the survey. These study findings imply that there is need for measures to be put in place to ensure that mothers access bed nets easily since access seems to translate into use.

## Conclusion

The study showed that the level of uptake of IPTp1 (at least one dose) was moderately high at 63.7 % while IPTp2 (at least two doses) was relatively low at 42.0 %. The IPTp2 coverage was far below the national target of 85 % in 2015. More than half, (58.6 %) of the respondents slept under an ITN the night before the survey. It was also shown that, about half of the respondents were knowledgeable about the use of IPTp tablets. The main factors that were associated with uptake of IPTp2 by pregnant women included education level of mothers and provision of free safe water at ANC clinics. The education level of mothers, ease of ITN access and parity of mothers were the main factors that were statistically significant for utilization of bed nets by pregnant women.

The district health team should consider intensifying health education campaigns about IPTp in the communities to improve the level of knowledge about IPTp among pregnant women. Emphasis should be put on explaining the purpose and the frequency of taking SP tablets for IPTp. Health workers should increase awareness and encourage mothers to continue sleeping under mosquito nets no matter how many pregnancies they have had. Health facilities should ensure that there is free safe water at ANC clinics at all times for mothers to swallow the SP tablets. The Ministry of Health should ensure that all ANC clinics are stocked with SP tablets at all times and that enough ITNs are available for distribution to health facilities.

## Abbreviations

ANC: antenatal care
DOT: directly observed treatment
ITN: insecticide-treated bed net
IPTp: intermittent preventive treatment in pregnancy
NGO: Non-government organization
PNC: postnatal care
SP: sulfadoxine-pyrimethamine
UMIS: Uganda malaria indicator survey
VHT: Village health team
WHO: World Health Organization
